# Polish infection control nurses’ job satisfaction and cooperation with their colleagues reflect how the value of infection control is appreciated by other health care workers: findings from surveys conducted before and during the COVID-19 pandemic

**DOI:** 10.1186/s13756-023-01284-2

**Published:** 2023-08-09

**Authors:** Dorota Jaślan, Jerzy Rosiński, Marta Wałaszek, Renata Majewska, Anna Szczypta, Jadwiga Wójkowska-Mach, Anna Różańska

**Affiliations:** 1 Chair of Microbiology, Jagiellonian University Medical College, ul. Czysta 18, Kraków, 31-121 Poland; 2 Institute of Economics, Finance and Management, Faculty of Management and Social Communication, Jagiellonian University, ul. Łojasiewicza 4, Kraków, 30-348 Poland; 3 Faculty of Health Science, University of Applied Sciences in Tarnów, ul. Mickiewicza 8, Tarnów, 33-100 Poland; 4 Chair of Epidemiology and Preventive Medicine, Jagiellonian University Medical College, ul. Kopernika 7, Kraków, 31-034 Poland; 5 Faculty of Medicine and Health Science, Andrzej Frycz Modrzewski Krakow University, ul. Herlinga-Grudzińskiego 1, Kraków, 30-705 Poland

**Keywords:** Infection control nurses, Infection prevention and control, Health care-associated infections

## Abstract

**Background:**

Infection prevention and control (IPC) is based on the activity of specialized, trained and highly qualified personnel, especially infection control nurses (ICNs). Effective implementation of IPC procedures demands close cooperation between IPC teams (IPCTs) and hospital personnel. Based on disturbing results on the epidemiology of health care-associated infections (HAIs) and compliance with preventive procedures, we suspect that cooperation between ICNs and different groups of hospital staff is poor.

The aim of this study was to assess the perceptions of ICNs working in Polish hospitals with regard to difficulties in working with various professional groups in the hospital, their organizational conditions, and their job satisfaction before and after the COVID-19 pandemic.

**Methods:**

The study was conducted twice, in 2014 and 2021, among ICNs working in Polish hospitals. The survey used an anonymous questionnaire designed by the authors.

**Results:**

In 2014, 183 ICNs participated in the study, and 175 ICNs participated in 2021. The respondents’ average age and seniority (duration of work as an ICN) were higher in 2021. Depending on the ward specialty, approximately 30–48.8% of the ICNs had difficulty cooperating with physicians. However, the ICNs declared better cooperation with nurses in various hospital wards and with other professionals. For some groups of hospital staff, there was a negative correlation between poor cooperation and ICNs’ job satisfaction. The job satisfaction data were disturbing; for example, more than half of the respondents considered changing jobs, and the lack of a sense of purpose in their work was declared by 29.7% of ICNs in 2014 and by 54.3% of ICNs in 2021.

**Conclusions:**

Our results suggest that infection prevention and control is not highly appreciated by health care workers and hospital management. Our study reveals difficulties in ICNs’ cooperation with hospital staff and managers in both 2021 and 2014, moderate job satisfaction, a high level of willingness to change jobs, and insufficient training in interpersonal skills and the implementation of changes. These findings clearly indicate an urgent need to introduce modern competence development systems in infection control beyond the scope of traditional training.

## Introduction

Modern medicine has an impressive array of tools to restore health and save lives, but hospital treatment, often involving invasive procedures, sometimes leads to the occurrence of infections, defined as health care-associated infections (HAIs) [1]. Infection of hospitalized patients is increasingly caused by multidrug resistant microorganisms (MDROs), which are difficult to treat and spread easily in health care facilities [2]. There have also been rapid changes in the epidemiology of infectious disease, such as the emergence of the highly contagious SARS-CoV-2. Under these circumstances, HAI control has emerged as a priority for modern health care. The planning, organization and implementation of HAI control in hospitals rests with the multidisciplinary hospital infection control team, whose key members are infection prevention and control professionals (IPCPs): medical doctors, nurses, or other health-related professionals who have completed a certified postgraduate IPC training course [3]. Infection control nurses (ICNs) control HAIs and play a key role in preventing them. The COVID-19 pandemic has made this specialization more necessary than ever.

The everyday work of ICNs includes collecting and analyzing infection data to make evidence-based decisions, educating health care professionals and nonmedical personnel in the field of infection prevention and control (IPC), isolating and treating infected patients to stop the spread of infectious diseases, and assisting in the development of action plans to minimize the effects of an outbreak in a hospital [4]. Attention to detail, the ability to work under pressure and excellent communication skills are of the utmost importance in this profession [5].

Disturbing data on HAIs in Poland have been published in recent years. One report [6] showed a six-times-higher incidence of surgical site infections after knee and hip replacement surgeries in Poland than the European average. Data on the incidence of surgical site infections after cesarean sections and on the use of antibiotics by women in the postpartum period indicate a lack of postdischarge surveillance in Polish hospitals [7, 8]. However, difficulties in effectively implementing HAI surveillance are not limited to Poland. Only fifteen European countries took part in the latest HAI-Net SSI ECDC program [9], and only twelve participated in the HAI-Net ICU [10].

The available research on hand hygiene in Polish hospitals reveals unsatisfactory knowledge and insufficient practical application on the part of hospital employees [11–13], but this is not a local or regional problem. Two reviews [14, 15] clarify the need for hand hygiene education both in countries with limited resources and in developed nations [14, 15].

In this context, we aimed to examine perceived difficulties in cooperation between the members of infection control teams, especially ICNs, and medical personnel engaged in the direct care of patients.

The aim of this study was to assess the perceptions of ICNs working in Polish hospitals with regard to difficulty working with various professional groups in the hospital, their organizational conditions, and their job satisfaction before and after the COVID-19 pandemic.

## Materials and methods

The study was conducted among nurses working in Polish hospitals as ICNs. The study used a questionnaire designed by the authors. In addition to questions characterizing the respondents in terms of age, gender, seniority (duration of work as a nurse) and place of work, we asked questions regarding their assessment of the scale of difficulties in cooperating with various groups of hospital employees as well as their job satisfaction, including their willingness to change jobs and their readiness to recommend the workplace to other nurses. Questions on the usefulness of specialized training of ICN candidates in a given area were also included in the questionnaire.

The study was conducted twice, in 2014 and in 2021. In 2014, an anonymous paper questionnaire was distributed through national and provincial ICN consultants, and in 2021, it was distributed as an online questionnaire. A link to it was also sent by the consultants. To assess any perceived difficulties and problems in ICNs’ cooperation with various professional groups and to assess the nature of organizational and institutional obstacles, we used a three-point Likert scale (low, moderate, high). To assess the degree of job satisfaction, we used a five-point Likert scale (definitely not, probably not, hard to say, probably yes, definitely yes). Questions about reasons for wanting to change jobs called for yes or no answers. Participation in the study was anonymous, so there is no information on how many people completed both surveys. In 2014, before distributing the questionnaire, we performed a pilot study in order to check respondents’ acceptance and proper understanding of questions. All studied variables were summarized using descriptive statistics. For categorical variables, frequencies are presented along with percentage distributions, while for quantitative variables, the means and standard deviations are presented in the case of normally distributed variables and medians and quartiles are presented otherwise. Aspects of job satisfaction expressed on a scale of 1 to 5 are also summarized by means and standard deviations. To compare the examined characteristics between 2014 and 2021, the chi-square test or Fisher’s exact test was used for variables expressed on a qualitative scale, the Kolmogorov‒Smirnov test was used for ordinal variables (assessment of the usefulness of training, severity of problems), and Student’s t test or the Mann‒Whitney test was used for quantitative variables. In addition, the level of problems related to cooperation with doctors and nurses in individual units was compared using the Friedman test for related couples. Kendall’s τ coefficient test was used to assess the strength of the relationship between the declared extent of problems and job satisfaction.

For the statistical analysis of the collected material, IBM SPSS (SPSS-Statistical Package for the Social Sciences, STATISTICS 24, Armonk, NY, USA) and Microsoft Excel (Microsoft Office 2016 Redmond, WA, USA) were used.

## Results

In 2014, 183 ICNs participated in the study, and 175 participated in 2021. The overwhelming majority of ICNs were employed in public hospitals in both phases of the study: 74% in 2014 and 77.7% in 2021. For both time points, the study did not reveal any differences in terms of the respondents’ gender, but there were significant differences by age, seniority as an ICN, and the size of the hospitals where the respondents worked. In 2014, the median age of the respondents was 48.7 years, rising to 51.6 years in 2021. Median seniority as an ICN was 23.8 years and 29.7 years, respectively. The median number of beds in hospitals where the respondents were employed was 447 in 2014 and 326 in 2021. The number of ICNs per hospital bed did not change (Table 1).

**Table 1:** Characteristics of the studied infection control nurses (ICNs)

Feature		2014 (n = 183)	2021 (n = 175)	p
Sex	female	183 (100.0%)	174 (98.4%)	0.489
	male	0 (0.0%)	1 (0.6%)	
Age		48.7 ± 6.87	51.6 ± 6.82	< 0.001
Province	Lublin	11 (6.0%)	10 (5.7%)	< 0.001
	Lesser Poland	27 (14.8%)	28 (16.0%)	
	Masovian	17 (9.3%)	30 (17.1%)	
	Subcarpathian	14 (7.7%)	14 (8.0%)	
	Silesian	56 (30.6%)	19 (10.9%)	
	other provinces	58 (31.7%)	74 (42.3%)	
Specialization	epidemiological nursing	160 (87.4%)	158 (90.3%)	0.392
	epidemiology	4 (2.2%)	0 (0.0%)	0.123
	hygiene and epidemiology	6 (3.3%)	5 (2.9%)	0.817
Year of obtaining certificate of trained ICN^*^		2008 (2005; 2010)	2011 (2008; 2016)	< 0.001
Seniority [duration of work as a nurse in years]		25.0 (17.0; 30.0)	30.0 (26.0; 35.0)	< 0.001
Work experience as an epidemiological nurse [years]		10.0 (6.0; 15.0)	12.0 (6.50; 17.25)	0.17
Hospital status	nonpublic	35 (20.0%)	25 (14.3%)	0.291
	private	10 (5.7%)	14 (8.0%)	
	public	130 (74.3%)	136 (77.7%)	
Number of beds in the hospital		392.0 (204.5; 619.8)	273.0 (180.0; 423.0)	< 0.001
Number of epidemiological nurses in the hospital		2.0 (1.0; 3.0)	2.0 (1.0; 2.0)	0.057

The table shows frequency (%), median (1st quartile; 3rd quartile), p – significance level

ICNs declared great difficulty in cooperating with hospital ward physicians at both time points without significant differences between the two years. The percentage of ICNs who reported major problems in cooperating with physicians was highest in surgical wards: 48.8% in 2014 and 44.4% in 2021. ICNs assessed their cooperation with physicians less negatively in intensive care units (ICUs) (major problems were indicated by 28.7% of respondents in 2014 and 2021) and with physicians in medical wards (32.1% in 2014 and 30.8% in 2021) (Table 2).

**Table 2:** Perceived usefulness of training and severity of problems in cooperation of infection control nurses with various groups of hospital employees

The usefulness of training in individual activities		2014	2021
Detection and registration of infections, data preparation and reporting	low	4 (2.4%)	16 (9.7%)
	moderate	48 (28.0%)	58 (35.2%)
	high	119 (69.5%)	91 (55.1%)
Cooperation with the microbiology laboratory, monitoring antibiotic usage and drug resistance	low	6 (3.6%)	7 (4.2%)
	moderate	55 (32.9%)	70 (42.1%)
	high	106 (63.5%)	89 (53.6%)
Hospital hygiene	low	0 (0.0%)	6 (3.6%)
	moderate	41 (24.4%)	55 (32.9%)
	high	127 (75.6%)	106 (63.4%)
Staff training – methods, effectiveness assessment	low	6 (3.9%)	12 (7.3%)
	moderate	65 (41.7%)	75 (45.7%)
	high	85 (54.5%)	77 (47.0%)
Interpersonal skills, motivating and implementing changes, cooperation with hospital staff	low	10 (7.0%)	18 (11.4%)
	moderate	70 (48.6%)	77 (48.7%)
	high	64 (44.5%)	63 (39.9%)
**Problems in**			
Cooperation with surgical ward medical staff	low	15 (9.1%)	16 (9.9%)
	moderate	69 (42.0%)	74 (45.6%)
	high	80 (48.8%)	72 (44.4%)
Cooperation with surgical ward nurses	low	59 (39.1%)	62 (39.5%)
	moderate	62 (41.1%)	63 (40.2%)
	high	30 (19.9%)	32 (20.4%)
Cooperation with intensive care unit medical staff	low	31 (22.8%)	27 (18.0%)
	moderate	66 (48.5%)	80 (53.3%)
	high	39 (28.7%)	43 (28.7%)
Cooperation with intensive care unit nurses	low	59 (46.8%)	64 (43.8%)
	moderate	39 (31.0%)	58 (39.7%)
	high	28 (22.2%)	24 (16.4%)
Cooperation with treatment ward medical staff	low	15 (9.0%)	21 (13.4%)
	moderate	99 (58.9%)	87 (55.7%)
	high	54 (32.1%)	48 (30.8%)
Cooperation with treatment ward nurses	low	58 (36.9%)	63 (40.4%)
	moderate	68 (43.3%)	71 (45.5%)
	high	31 (19.7%)	22 (14.1%)
Cooperation with the head doctor of infection control team	low	58 (35.8%)	74 (45.7%)
	moderate	50 (30.9%)	55 (33.9%)
	high	54 (33.3%)	33 (20.3%)
Cooperation with administration (accounting department, human resources department, purchasing department)	low	42 (25.8%)	46 (28.5%)
	moderate	84 (51.5%)	75 (46.6%)
	high	37 (22.7%)	40 (24.8%)
Cooperation with microbiological laboratory (hospital laboratory. external laboratory)	low	71 (45.0%)	73 (46.5%)
	moderate	48 (30.4%)	53 (33.7%)
	high	39 (24.7%)	31 (19.8%)
Cooperation with other employees (sterilization department, cleaning staff)	low	41 (27.3%)	59 (36.4%)
	moderate	89 (59.3%)	80 (49.4%)
	high	20 (13.3%)	23 (14.2%)
Cooperation with hospital management (directors: general, medical)	low	37 (22.9%)	57 (35.2%)
	moderate	75 (46.6%)	62 (38.2%)
	high	49 (30.4%)	43 (26.6%)

ICNs assessed cooperation with nurses working in hospital wards slightly better in 2021 than in the earlier study, but the difference was not significant: in surgical wards, 19.9% reported problems in 2014 and 20.4% in 2021; in intensive care units, the percentages were 22.2% and 16.4%, respectively; and in medical wards, the percentages were 32.1% and 30.8%, respectively. The differences in the frequency of responses indicating greater problems in cooperation with physicians than with other nurses working in hospital wards at both time points were statistically significant. The p value for differences in difficulties in cooperation between ICU job categories was 0.002, while in other categories, it was below 0.001.

The declarations of the ICNs in 2021 suggest that their cooperation with the head doctors from IPC teams, microbiology laboratories and hospital management improved (fewer respondents declared great difficulties in cooperation, Table 2), but the difference in responses was not significant.

The questionnaire also asked the ICNs to assess the usefulness of IPC training courses they attended in five areas (Table 2). Differences in the distribution of answers between the two time points were not statistically significant, but fewer respondents declared high usefulness in all five areas in 2021 than in 2014. At both time points, the fewest respondents declared that courses on interpersonal skills and on conducting staff training were useful (Table 2).

Regarding difficulties in their work, in 2021, the ICNs significantly more often indicated a lack of support from hospital management (10.4% in 2014 vs. 32.6% in 2021, p < 0.001) and a lack of IPC committee involvement in the surveillance of infections (27.3% in 2014 vs. 45.7% in 2021, p < 0.001). On the other hand, there were improvements in the areas of IPC committee decision-making, IPCP knowledge, and the delineation of IPCP responsibility (Table 3).

**Table 3:** Perceived difficulties in the work of infection control nurse respondents

Difficulties	2014 (n = 183)	2021 (n = 175)	p
Lack of support from hospital management	19 (10.4%)	57 (32.6%)	< 0.001
Lack of decision-making of the infection control committee	77 (42.1%)	39 (22.3%)	< 0.001
Low level of knowledge of infection control team members	68 (37.2%)	7 (4.0%)	< 0.001
Low level of knowledge of infection control committee members	16 (8.7%)	20 (11.4%)	0.398
Lack of involvement of the infection control committee in surveillance of infections	50 (27.3%)	80 (45.7%)	< 0.001
Lack of interest by medical staff in infection control	92 (50.3%)	105 (60.0%)	0.064
Reluctance of medical staff (wards) to take action for infection prevention	93 (50.8%)	88 (50.3%)	0.920
Lack of well-defined responsibility of infection control team members	89 (48.6%)	27 (15.4%)	< 0.001
Lack of well-defined responsibility of infection control committee members	26 (14.2%)	22 (12.6%)	0.650
Other difficulties (not precisely indicated)	69 (37.7%)	12 (6.9%)	< 0.001

Respondents at both time points assessed their job satisfaction similarly on a five-point Likert scale: general job satisfaction, mean 3.1 in 2014 and 3.0 in 2021; difficulties related to task performance, mean 3.3 in 2014 and 3.1 in 2021; salary, mean 2.1 in 2014 and 2.2 in 2021 (Table 4).

**Table 4:** Aspects of job satisfaction and intention to change jobs

Aspects of job satisfaction		2014 (n = 183)	2021 (n = 175)	p
Difficulty in completing tasks		3.1 ± 0.95	3.0 ± 1.06	0.732
*lack of data*		*15*	*0*	
Job (character) satisfaction		3.3 ± 1.03	3.1 ± 1.27	0.063
*lack of data*		*16*	*0*	
Salary satisfaction		2.1 ± 1.11	2.2 ± 1.14	0.406
*lack of data*		*13*	*0*	
**Job change**				
No		29 (43.9%)	81 (46.3%)	0.867
Yes, within the current unit		12 (18.2%)	27 (15.4%)	
Yes, to another hospital		25 (37.9%)	67 (38.3%)	
*lack of data*		*117*	*0*	
**Reason for wanting to change job**				
Salary		3 (8.1%)	52 (55.3%)	< 0.001
No development opportunities in the current job		20 (54.1%)	10 (10.6%)	< 0.001
Negative atmosphere at work. poor communication		7 (18.9%)	35 (37.2%)	0.043
Inconsistency with professional interests		12 (32.4%)	1 (1.1%)	< 0.001
Lack of opportunity to use the possessed skills and qualifications		0 (0.0%)	30 (31.9%)	< 0.001
Lack of sense of purpose in the work performed		11 (29.7%)	51 (54.3%)	0.011
Other reasons (not precisely indicated)		15 (40.5%)	10 (10.6%)	< 0.001
**Willingness to recommend the work of an epidemiological nurse**				
Definitely not		45 (31.0%)	35 (20.0%)	< 0.001
Probably not		66 (45.5%)	55 (31.4%)	
Hard to say		15 (10.3%)	36 (20.6%)	
Probably yes		18 (12.4%)	38 (21.7%)	
Definitely yes		1 (0.7%)	11 (6.3%)	
*lack of data*		38	0	

Fewer than half of the surveyed ICNs declared that they wanted to remain in their job (43.9% in 2014, 46.3% in 2021), but the reasons for their perceived need to change jobs changed significantly. The following were identified as unfavorable: low earnings, 8.1% in 2014 vs. 55.3% in 2021; lack of opportunity to use their skills and qualifications, 0% vs. 31.9%; and lack of a sense of purpose in their work, 29.7% vs. 54.3% (Table 4). Positive changes included a decrease in the percentage of ICNs who declared that they had no development opportunities (54.1% vs. 10.6%, p < 0.001) and a decrease in the percentage of ICNs who declared that the work was inconsistent with their professional interests (32.4% vs. 1.1%) (Table 4).

Analyses of correlations related to ICNs’ job satisfaction in 2021 showed that it became more difficult for ICNs to carry out their tasks if they had poor cooperation from the IPCT doctor (R = -0.220, p < 0.001) and other hospital employees (R = -0.211, p < 0.001). Furthermore, job satisfaction among ICNs decreased over time (R = -0.214, p < 0.001). Decreased job satisfaction among ICNs in 2014 was correlated with poor cooperation with the IPCT doctor (R = -0.235, p < 0.001), surgical ward nurses (R = -264, p < 0.001), hospital administration (R = -336, p < 0.001) and hospital management (R = -0.252, p < 0.001) (Table 5).

**Table 5:** Correlations between the perceived extent of problems and job satisfaction

Level of job satisfaction	2014			2021		
Difficulties in coopertatin with	Difficulty in completing tasks	Job satisfaction	Salary	Difficulty in completing tasks	Job satisfaction	Salary
Surgical department medical staff	0.001	-0.105	-0.098	-0.192^**^	-0.194^**^	-0.095
Intensive care unit medical staff	-0.137	-0.128	-0.049	-0.102	-0.093	-0.046
Treatment ward medical staff	-0.134	-0.101	-0.009	-0.121	-0.122	-0.027
The head doctor of infection control team	-0.087	**-0.235** ^**^	-0.165^*^	**-0.220** ^**^	-0.135^*^	-0.063
Surgical ward nurses	-0.029	**-0.264** ^**^	0.016	-0.162^*^	-0.076	-0.131
Intensive care unit nurses	-0.031	-0.184^*^	0.103	-0.114	-0.032	-0.077
Treatment ward nurses	-0.050	-0.170^*^	0.014	-0.156^*^	-0.084	-0.067
Administration (accounting department, human resources department, purchasing department)	0.006	**-0.336** ^**^	-0.048	-0.180^**^	-0.132	-0.086
Microbiological laboratory (hospital laboratory. external laboratory)	-0.113	-0.177^*^	-0.011	-0.180^**^	-0.148^*^	-0.101
Other employees (sterilization Department, cleaning staff)	0.005	-0.197^**^	0.118	**-0.211** ^**^	**-0.214** ^**^	-0.114
Hospital management (directors: general, medical)	-0.145^*^	**-0.252** ^**^	-0.122	-0.174^*^	-0.105	-0.096

*p < 0.05; **p < 0.01

## Discussion

We analyzed data on ICNs’ attitudes, their perception of cooperation with different groups of hospital staff, and their job satisfaction in 2014 and in 2021 during the COVID-19 pandemic. In this paper, the SARS-COV-2 pandemic serves as a time stamp, but we do not assess the strength and direction of its impact on our specific results. Clearly, however, the COVID-19 pandemic has had a negative impact on many areas of social and economic life, and it has brought more constraints and challenges to the health sector. Infection control is an integral part of modern health care, especially in hospitals. In Poland, modern procedures in this area date to the end of the 20th century, but a number of difficulties in implementing effective infection prevention programs in hospitals were observed just before the pandemic in association with the underestimation of its importance by medical staff [16]. This was supported by a study in 2016 [17] that showed that medical personnel perceive the activity of infection control teams, particularly ICNs, as a burden on the daily work of wards and that they are not respected by ward staff. The continuation of this phenomenon in 2021 is disturbing. The year 2021 was in the midst of the COVID-19 pandemic, so better cooperation might have been expected among all medical staff in addition to increased support and appreciation of infection control work, which is critical to the safety of both patients and staff. In contrast to this expectation, the respondents reported a lack of support from hospital management three times more frequently in 2021 than they did in 2014. In 2021, they also reported a lack of IPCP involvement in infection surveillance almost twice as frequently, and they were 20% more likely to report that medical staff lacked interest in it (60% of responses in 2021). It is difficult to explain these disturbing results. One reason may be that the ICNs did not have a good command of the skills needed to implement change and cooperate with ward personnel and managers. Compared to the responses related to substantive issues of IPC, our respondents were least likely to indicate that interpersonal skills training and staff training were highly useful. However, knowledge and skills without successful implementation are pointless. The ineffectiveness of personal protective equipment usage and hand hygiene training was confirmed by a study conducted during the pandemic in Poland’s largest clinical hospital [13].

The World Health Organization has prioritized the development of global guidelines on the essential elements of effective IPC programs, including the education of health professionals [18]. However, we examine competence development systems that go beyond the training and selection of development tools that depend on a specific competence, the level of development at the employee level, and the expectations of employers [19, 20]. Building a competence development system should be based on the participation of employees and management.

A separate research problem raised in this study was ICN job satisfaction, a phenomenon that has been studied quite often in ward nurses with regard to different potential factors [21–23]. Job satisfaction is often connected with an intention to leave the job. Nursing shortages have posed a challenge for some time in many countries. Nursing job satisfaction is a global concern because of its potential impact on patient safety. The work of ICNs also affects health care workers’ safety, as was especially evident during the COVID-19 pandemic. Our finding that only 44% (2014) and 46% (2021) of the surveyed ICNs declared willingness to continue working in their positions is truly disturbing and is of great concern in view of the average age of Polish ICNs (49 years in 2014 and 52 years in 2021), which clearly indicates rapid aging of this professional group and the lack of inflow of young ICNs to the Polish ICP system. Perhaps this unfavorable staff situation is related to the low satisfaction of Polish ICNs with their work, which is exacerbated by low wages. Psychological research has shown that as nurses’ job satisfaction increases, both their quality of life and the quality of nursing care improve [24]. Our statistics clearly show a relationship between reduced ICN job satisfaction and problems cooperating with IPCP doctors, ward nurses, administrative staff and hospital management.

## Conclusions

Our results suggest that infection control is not highly valued by health care workers and hospital management. Systemic efforts toward strengthening the position of ICNs should be urgently undertaken to increase their job satisfaction and encourage them to remain on the job. Our results also clearly indicate the need to implement modern IPC competence development systems that go beyond traditional training and suggest that ward staff and management should be included in this process.

## Data Availability

Data are available upon inquiry from Anna Różańska.
